# The contribution of a ‘whole of government’ smoke-free policy on the island of St Helena

**DOI:** 10.1080/16549716.2019.1681756

**Published:** 2019-11-07

**Authors:** Angela Mary Jackson-Morris

**Affiliations:** St Helena Government, Jamestown, Saint Helena

**Keywords:** Tobacco, smoking, government, policy, island

## Abstract

Under its Health Promotion Strategic Framework 2018–19 the St Helena Government prioritised action to address smoking and obesity to reduce a high non-communicable disease burden. The first tobacco control measure was a policy, ‘Smoke-Free Government’ (SFG), to create smoke-free public outdoor and indoor sites across all sites and services for staff and public users, abolish ‘official’ staff ‘smoking breaks’, and establish and promote community-wide cessation support. This paper assesses the perceived acceptability and preliminary impact of SFG in St Helena 2018–19. An online survey of government staff was undertaken 6 months post-SFG implementation to obtain insight into perceived impact, implementation, and acceptability. A population-wide health survey provided smoking prevalence and quit data prior to, and 11 months post-implementation. A majority of staff believed the policy contributed to reducing smoking, was generally observed, accepted, and entailed one or more positive effects, including reduced second-hand smoke exposure, increased quit attempts, and reduced disruption from ‘smoke-breaks’. Recommendations were consistent enforcement and expanded quit support. Population data for the SFG period indicated that smoking, and particularly daily smoking declined, quit intentions increased, and quit attempts almost doubled. The SFG policy appears to have contributed positively towards stronger tobacco control in St Helena in 2018–19.

## Background

Article Eight of the Framework Convention on Tobacco Control (FCTC) requires Parties to adopt effective national legislation for comprehensive smoke-free environments [1] Indoor public smoke-free legislation is the most implemented FCTC article [2] and is estimated to have averted 2.5 million deaths by 2013 [3]. The Article further urges Parties to create smoke-free environments in outdoor spaces where tobacco smoke exposure presents a hazard.

The number of jurisdictions enacting outdoor smoke-free legislation in specified public spaces is increasing, particularly at schools, hospitals, parks and beaches. Some countries have enacted national legislation incorporating specified outdoor areas [4], although provincial, state or municipal level laws are more common. Reduced smoking and second-hand smoke exposure (SHS), increased smoking quit rates, and reduced consumption have been reported across various settings [5–8]. Wider benefits include de-normalising, reduced youth initiation, and quit instigation [9–11].

A separate and intersecting area is a smoke-free workplace policy. In some countries these are included under national smoke-free legislation; elsewhere policies have been created by state or municipal ordinances for public-sector workplaces, and by private-sector organisations for their own staff. Most focus on indoor spaces, although some include outdoor sites and vehicles, and have been associated with reduced smoking prevalence, consumption, and quitting [12].

St Helena is a remote self-governing UK Overseas Territory in the South Atlantic, one of the 14 island territories spread across the Caribbean, South Atlantic, and Pacific, with historic and political ties to the UK [13] with a population in 2016 of 4,534 [14]. The island is geographically within the WHO ‘AFRO’ region, and although categorised in the World Bank’s ‘upper-middle income’ country band, the island depends on Overseas Development Aid for a sizeable proportion of national income [15].

Non-Communicable Diseases (NCD) account for a substantial burden of mortality, morbidity and health costs (on-island treatment as well as cost-intensive overseas referrals.) In 2018 a Health Promotion Strategic Framework (HPSF) was developed to support population behaviour change on key risk factors, by creating a more health-enabling environment [16]. Tobacco use is high at 24.2% of men and women (over 15 years), and very high rates among young men (50%) and women (46%) aged 20–29 years (Unpublished 2016 Census data.) Needs assessment early in 2018 identified common perceptions that: ‘Smoking is too easy’ (cheap and widely accepted) and ‘Smoking is everywhere’ and these were seen to make initiation easy and quitting difficult. Prior to 2018 island tobacco control primarily focused upon health education.

The HPSF included plans for various ‘MPOWER’ tobacco control measures [16], including legislation for packaging and advertising restrictions and tobacco taxation, however these entailed substantial development time and to deliver impact. A nearer-term opportunity was identified, building on the government’s influence and leadership. Government employs a quarter of St Helena’s adults, and public service delivery and employment sites occupy sizeable, prominent public spaces across the community (health, social care, education, administration, customs, justice, recreation, harbour, and emergency services). Indoor smoke-free legislation was enacted in 2012, yet smoking remained prevalent on outdoor sites and staff ‘smoking breaks’ were sanctioned.

The ‘Smoke-Free Government’ (SFG) policy offered a means to ‘lead by example’ to address the normative presence of smoking in the community by creating smoke-free zones on public sites, and to actively promote workforce health by prohibiting staff ‘smoking breaks’ and encouraging community-wide quitting. The policy catalysed plans to establish smoking cessation clinics, ‘brief intervention’ by clinicians to discuss smoking with patients, free nicotine replacement therapy (NRT), and social marketing to promote quitting and support.

## Methods

An online survey was undertaken 6 months after the policy began to obtain insight into perceived impact, implementation, and acceptability from the perspective of government staff. The response rate was 27% of staff with online access (21% of all government staff), including a proportion of smokers similar to the wider population. Additionally, to assess whether self-reported prevalence and quit rates had changed since ‘Smoke-Free Government’ was enacted, data were obtained from a population ‘Health and Lifestyles’ baseline survey undertaken prior to SFG (April 2018) and a year later, 11 months after SFG commenced. Validated GTSS Tobacco Questions for Surveys [17] were included and the survey was available online, on paper, and by face-to-face interviews to enable participation across a wide range of community members. The response rate comprised 9–10% of the total population each year (n = 417; n = 453), representative of gender, age, and residential area.

## Results

One-fifth of all government employees (almost one-third of staff online) participated in a survey 6 months following policy implementation. A majority of respondents (61%) believed the policy contributed positively to reducing smoking, and 68% perceived the policy had at least one positive effect. Reduced second-hand smoke exposure was most frequently perceived (67%), and 53% believed the policy had increased quit attempts and reduced smoking. Perceived reduced disruption to the workday by outlawing ‘smoking breaks’ was identified by 42%. Other perceived effects were fitter or healthier staff, staff walking further to smoke off-site at lunchtime, and reduced cigarette litter.

Sixty-two percent of responders believed the policy was generally observed by the majority of staff/public. Implementation was perceived to be weaker in specific areas of a small number of sites and teams, particularly those with flexible or peripatetic working patterns. Whereas a majority (65%) of staff did not identify any practical issues or difficulties, 35% specified areas to be strengthened. More consistent enforcement by managers where implementation was weaker was the most frequent recommendation. Clearer guidance for off-site smoking, how to request compliance, and how to support stressed staff or service-users was also suggested.

A majority of responders (58%) considered the policy to be generally accepted by most staff/the public. However, 24% did not believe the policy was well accepted and 18% were equivocal. Comments indicated that acceptance was more limited among specific parts of the workforce and in relation to a small number of sites. Most responders’ views were the same as prior to the policy (71%); however, 21% said they had come to view the policy more positively over time. A large majority (76%) believed further actions to reduce smoking were needed. Most suggested strengthening SFG, particularly implementation at ‘hotspots’ and expanding quit support. Stress support and training were also highlighted as an issue made more apparent by SFG.

The 2019 population survey occurred 11 months after ‘Smoke-Free Government’ commenced. During this period self-reported smoking decreased from 21% (14% daily) to 12% (8% daily), smokers ‘seriously thinking about quitting’ increased from 22% to 39% and 24 h quit attempts increased 11%.

## Discussion

The ‘Smoke-Free Government’ policy was perceived to have generated tangible health benefit and to have contributed to stronger tobacco control in the community. The policy, accompanied with cessation support and promotion, was perceived to have contributed to increased quit intentions and attempts, reduced consumption, and may have contributed to the reported decline in self-reported smoking between 2018 and 2019. It is not possible, however, to identify the relative contributions of the policy, the quit support and wider quit promotion over the period.

Key advantages were that SFG could be implemented within the existing government system and structures, it was low cost and related to the government’s workforce and public sites under its jurisdiction. It could therefore proceed earlier than other planned measures that required greater lead-in time and multi-sectoral ‘buy-in’. Governmentwas able to leverage its prominent role and influence to ‘lead by example’. It may be expected that this would be the case in a close-knit island community with a small population, and this example suggests that a ‘whole of government’ policy, integrating cessation support and promotion, could contribute positively to tobacco control in island settings, as part of the tobacco control policy mix being developed for island states [18, 19]. Local and regional governments serving defined populations across multiple public sites may also achieve benefit from an SFG approach. Globally, the public sector constitutes the largest employer [20], public services and sites are highly used and occupy substantial, prominent community spaces, thus the potential is substantial.

A proportion of staff either were equivocal or did not support the policy. This may partly relate to the policy as a ‘top-down’ measure that most affected those who were most tobacco-dependent. The findings added impetus to further develop quit support and strategies to engage and motivate smokers to consider quitting. However, a majority of staff believed the policy was generally accepted and observed by most staff and the public and over one fifth said their view had become more positive. This suggestion of an evolution towards increased support adds to research that shows similar shifts regarding smoke-free indoor policies [21,22] and standardised packaging [23]. Research suggests that smoke-free policies in one sphere may encourage support for an extension to other areas [24,25], and policymaker confidence to undertake further measures may grow following evidence that policies were well received and not destabilising [26]. St Helena policymakers cited their perception that the policy had become generally viewed as a positive contribution to community health as one factor along with the availability of cessation support, that encouraged their support for further measures, including passage of WHO-recommended level tobacco taxation in August 2019 [27].

## Paper context

Government workforces comprise sizeable proportions of their populations; government services and sites are used by large numbers of people, and government sites occupy substantial geographical zones across communities. Outdoor public site and workforce smoke-free policies have – separately – shown positive contributions to reduced smoking, increased quitting and reduced second-hand smoke exposure. This island example combined approaches. Data suggest such a policy can contribute to positive population tobacco control outcomes.
